# Electrophysiological Studies on The Dynamics of Luminance Adaptation in the Mouse Retina

**DOI:** 10.3390/vision1040023

**Published:** 2017-10-17

**Authors:** Anneka Joachimsthaler, Tina I. Tsai, Jan Kremers

**Affiliations:** 1 Department of Ophthamlology, University Hospital Erlangen, 91054 Erlangen, Germany; 2 Department of Biology, Animal Physiology, FAU Erlangen-Nürnberg, 91058 Erlangen, Germany

**Keywords:** electrophysiology, ERG, mouse, adaptation

## Abstract

To date, most studies involving in vivo electroretinography in mice are performed on steady state adapted animals. In this study, we focused on the dynamics of adaptation to high and low light levels in the mouse retina. Two flash electroretinogram (ERG) protocols and one flicker ERG protocol were employed. In the two flash ERG protocols, the animals were adapted to either 25 or 40 cd/m^2^ white light and ERGs were recorded for up to 15 min of adaptation. Afterwards, flash ERGs were recorded for up to 45 min of dark adaptation. Amplitudes of the flash ERG increased during light adaptation, while implicit times of the different wave components decreased. During subsequent dark adaptation, the amplitudes further increased. The increase in a-to-b-wave ratio indicated adaptational processes at the photoreceptor synapse. In the flicker ERG protocol, the responses to 12 Hz sinusoidal luminance modulation during the adaptation to 25 cd/m^2^ and a 1 cd/m^2^ mean luminances were recorded. The amplitudes of the first harmonic components in the flicker protocol decreased during light adaptation but increased during dark adaptation. This is at odds with the changes in the flash ERG, indicating that adaptation may be different in different retinal pathways.

## 1. Introduction

The electroretinogram (ERG) is a non-invasive in vivo electrophysiological method that enables the analysis of physiology and the functional integrity of the retina. In most ERG studies in the mouse, some time is allowed for the eye to adapt to a specific background light before measurements begin, so that a ‘steady state’ or constant mode of operation is reached [1,2,3,4,5,6,7,8]. Adaptation is an important dynamic phenomenon that adjusts retinal responsivity to obtain optimal transmission of visual information to the different brain areas. The dynamic process of activity alteration after a change in mean luminance and/or chromaticity is thus an important feature of retinal physiology that has yet to be fully investigated. On a cellular level, there is evidence that adaptation processes take place at different retinal locations. In the mouse retina, for example, adaptation has been localized in the outer segments of the photoreceptor cells [9,10,11,12], at the photoreceptor synapse [13], at the gap junctions between photoreceptors [14] and in the inner retina [15,16,17,18].

In comparison, data on the physiological manifestations of adaptation-related changes using the ERG are more readily available. Several such studies have been performed on humans and non-human primates [19,20,21,22,23,24]. Collectively, they report that the amplitudes of flash or flicker ERGs increase during both light and dark adaptation. Peachey et al. [22,23] found the implicit time of the b-wave to decrease during light adaptation and the implicit time of the flicker ERG to decrease during both light and dark adaptation by about 3 ms. McAnany and Nolan [19] revealed that the implicit time of the ERG to sine-wave modulation also decreased slightly during light adaptation, even though the phase of the first harmonic remained constant. Similarly in canines, Maehara et al. [25] found an increase in the b-wave amplitude and a decrease in the b-wave implicit time during dark adaptation. After around 30 min, both parameters reached a plateau and did not change while the animals were further dark adapted.

Although rodents often are the animal models of choice in vision research, the information about the in vivo physiological dynamics of adaptation is still limited. Peachey et al. [26] found an increase in amplitude and a decrease in implicit time of the mouse cone-driven flash ERG during the first 6–10 min of light adaptation. Similar changes were also found by Brown et al. [27] and Cameron and Lucas [28] in mice and Bui and Fortune [29] in rats. Although there are studies that describe the changes in cone or rod ERGs after bleaching of the retina using paired flash stimuli [30,31,32], there are not many data about ERG responsivity changes during dark adaptation that are recorded with single flash ERGs or flicker ERGs. Li et al. [33] found an increase in amplitude and a decrease in latency in rodents during dark adaptation, which is similar to the results in the study of Maehara et al. [25] in canines.

The aim of the present study was to analyze, in detail, the dynamics of ERG response changes during adaptation to incremental (photopic) and decremental (scotopic) light levels in mice. In contrast to previous studies, where the adaptation dynamics to either incremental or decremental light levels were investigated, we present for the first time, data from the two simultaneously, in identical individual animals and even within one recording session. In contrast to previous studies, our data therefore enable a comparison of the different adaptation dynamics. Changes of several flash ERG components that are thought to originate from activity involving different retinal cell types were tracked during the different adaptation protocols. Furthermore, we studied alterations in the sinusoidal flicker ERG during adaptation to increments and decrements of mean luminances.

## 2. Results

### 2.1. Adaptation Kinetics of the Flash ERG

As mentioned in the methods, two different flash protocols—one with a dimmer background for a longer duration of light adaptation (‘protocol 1’, Figure 7A; 25 cd/m^2^ for 15 min), and the other with a brighter background for a shorter time of light adaptation (‘protocol 2’, Figure 7B; 40 cd/m^2^ for 5.5 min)—were used to examine the kinetics of light and dark adaptation in the mouse ERG.

### 2.2. Light Adaptation

**Figure 1:**
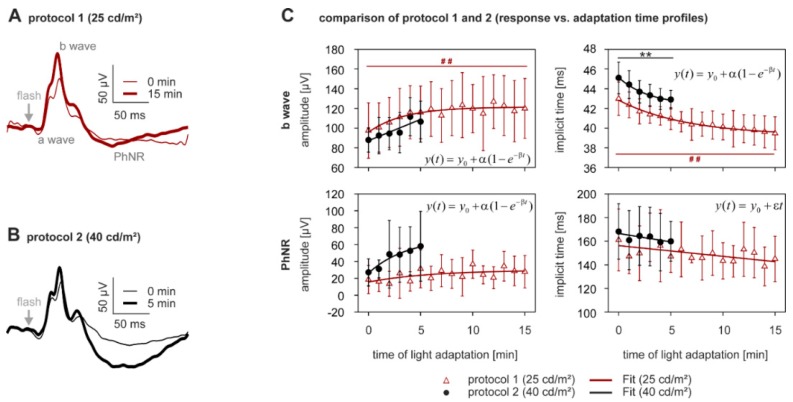
Light adaptation—a- and b-wave analysis of the photopic flash electroretinogram (ERG). (**A**) Subject averaged flash ERGs directly (0 min; thin curve) and 15 min (thick curve) after the onset of a 25 cd/m^2^ adapting light; (**B**) As in (A) directly (0 min; thin curve) and 5 min (thick curve) after the onset of a 40 cd/m^2^ adaptating light; (**C**) B-wave (upper plots) and photopic negative response (PhNR) (lower plots) amplitudes (left plots) and implicit times (right plots) as a function of adaptation time to photopic adaptation (red open triangles: 25 cd/m^2^; black closed circles: 40 cd/m^2^; means ± s.d.). The data are fitted by the functions described in the insets of the plots. n_25 cd/m_^2^ = 10, n_40 cd/m_^2^ = 9. The large variability in the PhNR amplitudes is caused by the interference of respiration movements on the ERG signal.

Flash ERG responses, recorded directly after the onset of photopic adapting light, already showed the conventional photopic flash waveform, composed of a small initial negativity (a-wave), followed by a larger positivity (b-wave), and a photopic negative response (PhNR) (Figure 1A,B and Figure 8B). The a-wave was very small (<10 µV), typical of mouse photopic flash ERGs, and was therefore not further analyzed. Directly after light onset, the components were rather small in amplitude compared to those recorded during steady state light adaptation. In the subsequent light adaptation period, the amplitudes showed a recovery.

The waveforms of ERGs recorded in protocol 1 (adaptation to 25 cd/m^2^) possessed larger b-waves and the PhNR components, after 15 min of light adaptation, compared at time 0 (baseline) (Figure 1A). Statistical analyses (Table 1) confirmed that the b-wave amplitude increase seen over the light adaptation period was significant (+21.8 ± 18.8% mean ± sd, *p* < 0.0005, Figure 1C upper left plot, open triangles), and that it reached a plateau after about 7 min of adaptation. The PhNR change during light adaptation, however, did not reach significance (Figure 1C, lower left plot), due to a high standard deviation. Likewise, corresponding implicit times of the b-waves significantly decreased with increasing light adaptation (−7.7 ± 4.2%, *p* < 0.0005, Figure 1C upper right plot, open triangles), whereas the timing changes of the PhNR were not significant (Figure 1C lower right plot). 

Light adaptation ERG measurements with protocol 2 (40 cd/m^2^; Figure 7B for averaged waveforms) are shown in Figure 1C (closed circles). Although measured over a shorter adaptation period, the b-waves showed similar changes to those recorded with the dimmer background, in protocol 1. However, the increase in b-wave amplitude was not significant (+22.3 ± 22.4%, *p* < 0.05, Figure 1C upper left plot), but it decreased significantly in its time to peak (−4.8 ± 1.8%, *p* < 0.0005, Figure 1C, upper right plot). Even though the PhNR exhibited a stronger light adaptation-dependent increase in size while adapting to 40 cd/m^2^, large variability in this component meant that the PhNR amplitude change was not significant (Figure 1C, lower left plot). Also, the decrease in the PhNR implicit time was not significant during the observed time period of light adaptation (Figure 1C, lower right plot). Parameters describing the fits of the flash ERG components as a function of light adaptation time are provided in Table S1.

To study if the 25 and 40 cd/m^2^ backgrounds influenced the ERG components differently, the responses at t = 0 min, and the changes in ERG components during the first 5 min of light adaptation, were compared. Statistical analyses (Table S2) revealed no differences in b-waves and PhNR alterations between the two protocols, after Bonferroni correction.

**Figure 2:**
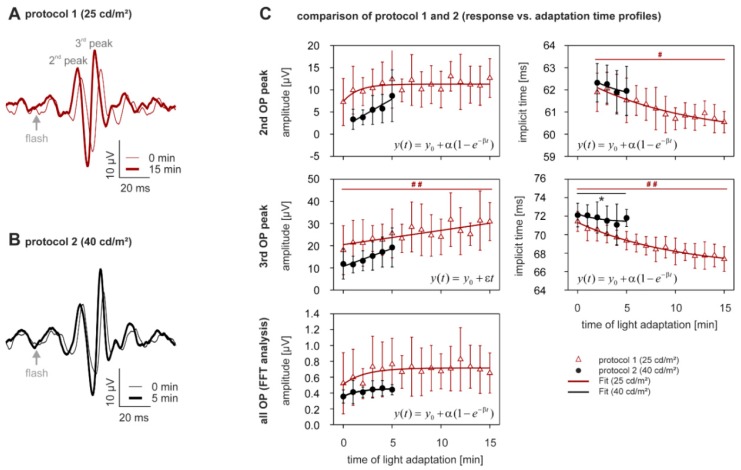
Light adaptation—OP analysis. (**A**,**B**) for subject averaged OPs in the same format as Figure 4. Subject averaged (**C**) amplitudes for the second and third OP waves and for the general OP in the frequency domain vs. time of photopic adaptation (same format as in Figure 4). n_25 cd/m_^2^ = 10, n_40 cd/m_^2^ = 9.

As for the oscillatory potentials (OPs), the amplitudes of the total response and of the second and third OP wavelets also appeared to increase with increasing exposure to both levels of photopic adapting light (Figure 2A–C left plots). Surprisingly however, only the amplitude increase of the third OP peak in protocol 1 (25 cd/m^2^ for 15 min) was statistically significant (+70.6 ± 57.9%, *p* < 0.0005, Figure 2C, open triangles), even though the average increase of the third peak in protocol 2 was greater (+102.9 ± 127.7%, *p* < 0.05, Figure 2C, closed circles). This was perhaps because only the third peak continued to grow, while the other components reached a plateau after 5 to 10 min of exposure to the adapting light. Corresponding implicit times of the OP parameters decreased during light adaptation (Figure 2C right plots). The decrease was significant for the second peak in protocol 1 (−1.7 ± 1.7%, *p* < 0.005, Figure 2C, open triangles) but not in protocol 2. The change in the implicit time of the third peak was significant in both protocols (protocol 1: −5.3 ± 1.8%, *p* < 0.0005, Figure 2C, open triangles; protocol 2: −0.7 ± 2.5%, *p* < 0.005, Figure 2C, closed circles).

The comparison of the t_0_-values exhibited no differences between the two protocols after Bonferroni correction. Moreover, all OP parameters changed similarly during the first 5 min of light adaptation in both protocols (Table S2).

### 2.3. Dark Adaptation

As mentioned in the methods, the scheme of ERG measurements during the dark adaptation period was different for the two protocols (protocol 1: recordings every 0.5–1.0 min; protocol 2: recordings every 1.0–2.0 min). Moreover, the dark adaptation period was longer in protocol 2 (up to 44 min instead of 27 min for protocol 1). This was possible because the light adaptation period was shorter in protocol 2, leaving more time for tracking dark adaptation before the animal woke up.

**Figure 3:**
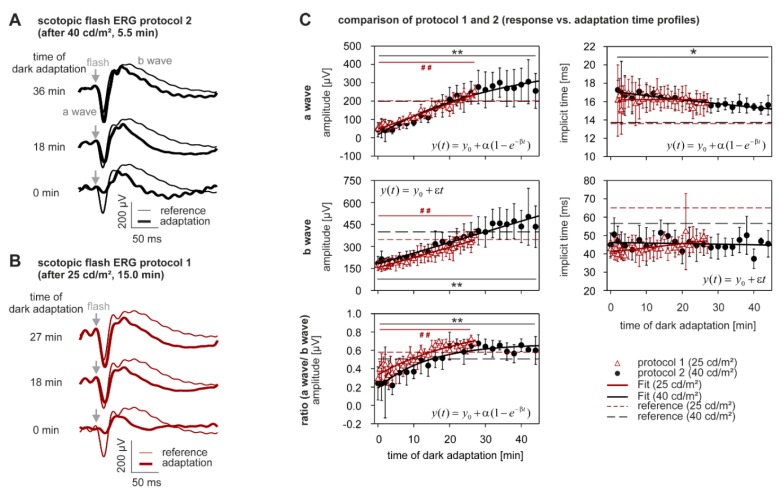
Dark adaptation—a- and b-waves. (**A**,**B**) Thick curves: scotopic flash ERGs (subject averaged) at different times after offset of the photopic adaptation. Thin curves: reference scotopic flash ERG recorded prior to photopic adaptation for comparison; (**C**) Amplitudes and implicit times vs. time of dark adaptation. The lower left plot shows the ratio of a- to b-wave amplitudes as a function of adaptation time. The dashed lines are values obtained from the reference measurements. n_after 40 cd/m_^2^ = 9, n_after 25 cd/m_^2^ = 5.

Of the scotopic ERG components, the a-wave, b-wave and OPs were analyzed. Figure 3A,B shows the ERGs from dark-adapted protocols 1 and 2, respectively. Although the scotopic responses recorded directly after light offset were small in comparison to the scotopic reference measurement recorded at the beginning of the session, they had the typical morphology of a scotopic ERG waveform. From the waveforms (Figure 3A,B) and plots of the scotopic a- and b-waves as a function of dark-adaptation time in Figure 3C (upper left panels), it can be seen that the initially small amplitudes of the a- and b-waves steadily increase directly after offset of the adapting light. Particularly, the growth of the a-wave with dark-adaptation time (i.e., the difference between maximum amplitude and baseline) was stronger than the growth of the b-wave. Indeed, a statistical comparison of their maximal changes to the scotopic baseline (Table 2) revealed that the increase in a- and b-wave amplitudes in both protocol 1 (Figure 3C; open triangles) and protocol 2 (Figure 3C; closed circles) were significant (a-wave: +309.3 ± 50.6% and +840.4 ± 571.0%, respectively, b-wave: +100.0 ± 38.7% and +126.8 ± 64.3%, respectively; all *p* < 0.0005). In any case, the amplitudes of the a- and b-waves reached a plateau that was close to the reference value (dashed lines) after around 30 min of dark adaptation. 

Since the initial amplitudes of the a-waves were smaller than those of the b-waves, we would expect a systematic change in the ratio of a- to b-wave amplitudes. The lower left plot in Figure 3C displays this ratio as a function of dark adaptation time, and shows that the ratio also increased significantly (+114.3 ± 45.6%, *p* < 0.0005 for dark adaptation following 25 cd/m^2^ light adaptation afterprotocol 1, open triangles; +301.9 ± 277.2%, *p* < 0.0005 after protocol 2, closed circles). The ratio reached a plateau about 20 min after the background light was turned off.

The implicit times of the a- and b-waves as a function of dark adaptation time are provided in the right-hand panels of Figure 3C. In comparison to the amplitudes, corresponding implicit times of the a- and b-waves did not change as strongly. Moreover, the implicit times might not have reached the level of the reference even after 36 min of dark adaptation. After this period, the a-wave trough still occurred about 2 ms later, and the b-waves peaked about 10 to 15 ms earlier, compared to the reference. This may indicate that very slow adaptation processes may influence the implicit times. In fact, during the 23 min of dark adaptation in protocol 1, implicit times of the a- and b-waves did not change significantly. In protocol 2, the a-wave implicit time showed a significant decrease of about 2 ms (−7.0 ± 14.2%, *p* < 0.005, open triangles, Figure 3C). The b-wave peak time, on the other hand, did not significantly change, even after more than 40 min of dark adaptation in protocol 2. A summary of the fitting parameters is given in Table S3.

The dark adaptation-response profiles of the ERG components in protocols 1 and 2 were further compared, by analyzing the absolute values at time point t = 0 min, and the change in amplitudes and implicit times, during the first 26 min of dark adaptation (a summary of the comparison is given in Table S4). This analysis revealed no significant differences between the two protocols for any of the evaluated parameters.

**Figure 4:**
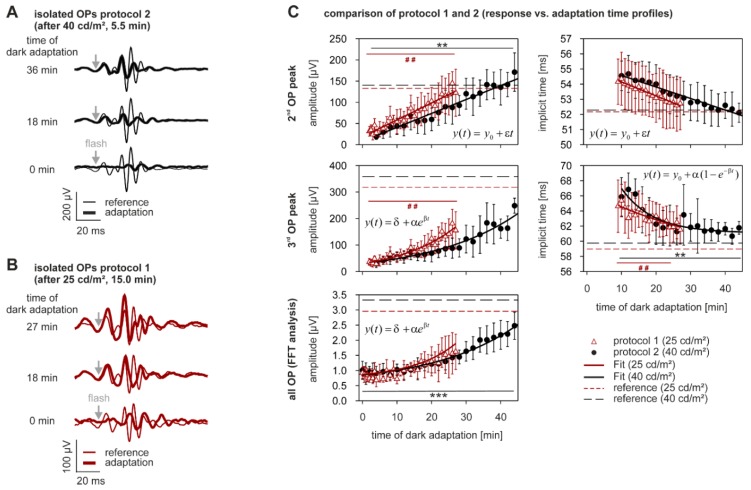
Dark adaptation—oscillatory potentials. (**A**,**B**) OPs, shown in the same format as Figure 6. (**C**) OP amplitudes and implicit times vs. time of dark adaptation (same format as in Figure 6). n_after 40 cd/m_^2^ = 9, n_after 25 cd/m_^2^ = 5.

The OPs of the scotopic flash ERGs were analyzed in a similar manner to those of the photopic ERG. The results are displayed in Figure 4. Panel A of the figure shows the isolated OPs recorded in protocol 2 (40 cd/m^2^) after 0 min, 18 min and 36 min of dark adaptation—each superimposed on those obtained with the reference measurement. The OPs were initially small and slow, but increased in size and decreased in their time-to-peaks, during dark adaptation. Different peaks of the OPs, however, showed distinct changes. After 36 min of dark adaptation, only the second peak appeared to match the reference waveform. Even after more than 40 min of dark adaptation, the other OP wavelets were still smaller than those of the reference response, indicating very slow adaptation processes in the OPs.

Similar effects were found with the scotopic OPs after offset protocol 1 adaptation (25 cd/m^2^; Figure 4B). The OPs were also small and delayed directly at the start of the dark adaptation. Again, different OP peaks showed distinct dark adaptation changes, as they did for protocol 2.

These descriptions were also manifested in the dark adaptation response functions for each OP peak (Figure 4C; see also Table 3). The statistical analysis revealed significant increases in amplitudes of the second (protocol 1: +321.9 ± 76.2%, *p* < 0.0005, open triangles; protocol 2: +588.2 ± 239.7%, *p* < 0.0005, closed circles) and third (protocol 1: +272.2 ± 85.5%, *p* < 0.0005, open triangles; protocol 2: +435.0 ± 170.6%, *p* < 0.05, closed circles) OP peaks, for both protocols. When the growth of the amplitude of the maximum OP response in the high frequency range after Fourier transformation was analyzed, it was only significant for protocol 2 (+124.4 ± 57.5%, *p* < 0.0005, closed circles).

Despite significant increases in the second and third OP peaks, only the implicit time of the third OP peak showed a significant decrease (protocol 1: −2.4 ± 4.6%, *p* < 0.0005, open triangles; protocol 2: +1.7 ± 4.4%, *p* < 0.0005, closed circles). The statistical analysis also revealed no significant differences for y(t_0_)-values or the change in parameters in the first 26 in of dark adaptation, between the two groups (Table S4).

### 2.4. Dynamics of Adaptation in the Flicker ERG

**Figure 5:**
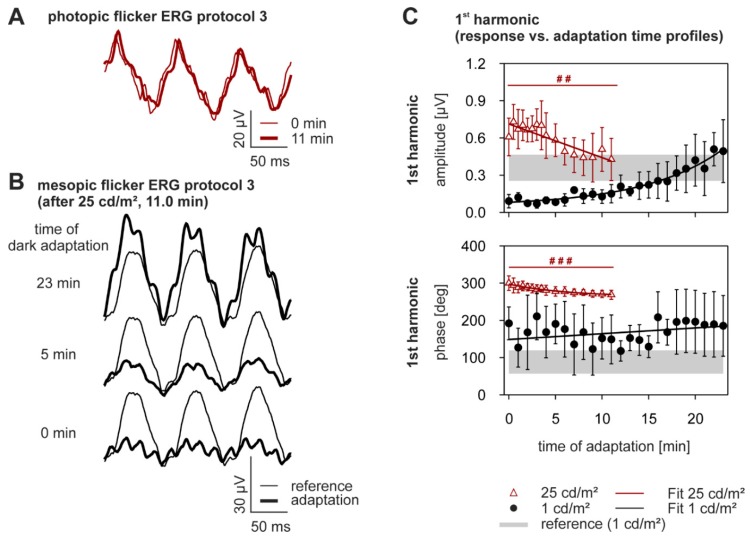
Light and dark adaptation recorded with the flicker adaptation protocol. (**A**) The flicker ERG response at 0 (thin curve) and 11 min. (thick curve) after onset of the 25 cd/m^2^ mean luminance; (**B**) Thick curves represent flicker ERGs at 0, 5 and 23 min after the onset of the 1 cd/m^2^ mean luminance. Thin curves represent reference measurements at 1 cd/m^2^ obtained at the beginning of the session; (**C**) Amplitudes (upper plot; means ± SD) and phases (lower plot) of the fundamental component as a function of adaptation time (red open triangles: 25 cd/m^2^; black closed circles: 1 cd/m^2^). The gray zone is the 95% interval of the reference measurements. n_light adaptation_ = 8, n_dark adaptation_ = 8.

In addition to the two flash protocols, the influence of adaptation to photopic and mesopic light levels on the flicker ERG was studied. The responses to the photopic flicker (25 cd/m^2^, 12 Hz sinusoidal modulation, 100% Michelson contrast) are displayed in Figure 5A. During the first 11 min of photopic adaptation, the ERGs hardly decreased in amplitude and shifted slightly in phase. After the mean luminance was reduced to 1 cd/m^2^, the flicker response became very small (Figure 5B), after which it grew steadily during the following 23 min of adaptation to the low luminance. These changes are reflected in the amplitude and phase plots of the fundamentals components at 25 and 1 cd/m^2^ as a function of adaptation time (Figure 5C, statistical analysis in Table 3). In fact, the amplitude decreased significantly by 25.8 ± 39.3% during the adaptation to 25 cd/m^2^ (*p* < 0.005, Figure 5C, open triangles) and the phase decreased by 8.7 ± 7.2% (*p* < 0.0005, Figure 5C, open triangles). The subsequent amplitude increase during the 23 min of adaptation to 1 cd/m^2^ was much greater in comparison (+438.1 ± 304.4%, Figure 5C, closed circles). However, the phase remained almost constant during adaptation to 1 cd/m^2^ (+51.1 ± 159.2%, Figure 5C, closed circles), which was slightly larger than the phase in the reference measurement. Due to the low signal-to-noise ratio of the recordings at 1 cd/m^2^ mean luminance, no statistics could be performed on the dark adaptation data. Interestingly, the fundamental’s phase changed at about 150 degrees, directly after the mean luminance was increased or decreased. A summary of the fits describing the flicker ERG parameters as a function of adaptation time to high and low light levels, is given in Table S5.

## 3. Discussion

In the present study, we examined the influence of adaptation to high and low luminance levels in the mouse retina using electroretinography. Until now the influence of adaptation was mostly studied on steady state responses. Adaptation is a complex process that may alter the retinal mode of operation at multiple stages. As a result, the responsiveness of the retinal mechanisms (that are reflected by the different ERG components) may display different adaptation-dependent dynamics. Therefore we chose to study the amplitudes and timings of different flash and flicker ERG components in detail.

Part of the adaptation effects shown in the present study might be caused by pigment bleaching. As far as we know, there are not many data on pigment bleaching in mice. In humans, it was found [34] that with a 3000 td mean retinal illuminance, 90% of the rhodopsin pigment remains unbleached (i.e., 10% is bleached). With a fully dilated pupil, this would correspond to about 60 cd/m^2^ luminance. Assuming that the mouse and human eyes are isometric (which is approximately true for these purposes), at 40 cd/m^2^ luminance, less than 10% rhodopsin will be bleached. The effects of bleaching on the ERGs will be even less when a 25 cd/m^2^ luminance is used or when the ERG signals are driven by the cones. We therefore conclude that the adaptation effects shown in the present study are mainly caused by neuronal mechanisms.

### 3.1. Time Constants of Adaptation

ERGs to white flashes were measured during light and dark adaptation protocols. In addition, the changes in ERG responses to sinusoidal luminance modulation were measured during adaptation levels that were different by a factor of 25. These studies demonstrated that the changes in flash and flicker ERGs during adaptation to high light levels were fast compared to those during dark adaptation (flash ERGs) or to low light levels (flicker ERGs). The changes to high light levels reached a plateau around 10 min after the onset of the light. This time was about 30 min or more for dark or low light level adaptation. This trend was evidenced for both amplitudes and timing parameters (implicit times or phases). Even though the time constants differed strongly between adaptation to high and low light levels, the responses that were recorded directly after background light on and offsets, directly showed the morphology of cone- and rod-based flash ERGs, respectively, without further subsequent changes in the ERG waveform. We therefore conclude that a change in background illumination (i.e., on or offset of background illumination) immediately leads to a shift between cone- and rod-driven responses.

### 3.2. Amplitudes

In the present study, the effects of adaptation to high light levels and to dark backgrounds were studied in the same session and on an identical group of animals. In comparison to previous studies on either one or the other adaptation type showing a similarly gradual amplitude increase in the flash ERGs during light (rats: [29], mice: [26,35], humans: [20,21,24]) and during dark adaptation (rodents: [33] and canines: [25]), our study demonstrates this to be the result of an instantaneous amplitude decrease upon the increase of background luminance. However, the amplitude increased further after the background illumination was decreased. Furthermore, our detailed analysis of the OPs revealed the second and third peak to increase with different time constants during dark adaptation. Specifically, the third OP peak increased much more slowly than the second OP peak. To our knowledge, this feature has not previously been described. Differences in the adaptation kinetics of the different OP wavelets might imply that they are generated by different inner retinal networks. This was first suggested by Wachtmeister [36], and is further supported by a recent study in rats showing different OP peaks to react differently to methanol intoxication (Liu et al. [37]). In regards to the influences of adaptation on sinusoidally-modulated flicker ERG responses, we show, for the mouse, that its first harmonic amplitudes decrease during adaptation to high light levels, whereas they increase during adaption to low light levels. The response amplitude decrease after light adaptation is at odds with the changes in the flash ERG components, where the component amplitudes increase, indicating that adaptation may be different in different retinal pathways. Furthermore, the amplitude decrease at high light levels is in contrast to the increase found in human flicker responses [19,22,23]. The discrepancy in our results may reflect differences in retinal physiology between humans and mice. Another explanation could be in relation to differences in our adaptation protocol. That is, that the photopic adaptation in the human subjects was studied after an extended period in the dark, whereas in our experiments, the animals’ ‘dark-adaptation states’ in this case, were to 1 cd/m^2^. Possibly, the difference in complete dark adaptation and a mean luminance of 1 cd/m^2^ (which is low mesopic or high scotopic) is sufficient to bring the retina into a different state of operation. It is known that rod-driven signals are transmitted through different pathways in these light levels [38,39].

### 3.3. Implicit Times

In agreement with previous studies in rodents [26,29,33], canines [25] and humans [20], we demonstrated that the implicit times of the flash ERGs decreased during light adaptation as well as during dark adaption. As proposed above for their corresponding amplitudes, the sudden increase after background light onset and subsequent decrease in implicit times over both light/dark adaptation periods is probably also a general recovery after an instantaneous change in implicit time following the alteration in adaptation level. 

The flicker ERG also showed a decrease in the fundamental phases during the adaptation to high light levels. Similar to the shift in the implicit time of flash ERG components after the onset of the adapting light, the phase of the first harmonic response changed instantaneously after the change in mean luminance. This large phase change is possibly caused by a change from cone- to rod-driven responses and vice versa. Recently, we indeed found that the rod-driven responses are particularly large at luminances below approximately 7 cd/m^2^ and mainly cone-driven for higher mean luminances [40]. Again, the phase changes described here for mice, are not consistent with previous findings for human subjects. McAnany and Nolan [19] found that the human ERG response phase of the first harmonic component did not change during high light level adaptation, but rather, there was an increase in phase for the second and third harmonic. Peachey et al. [22,23], on the other hand, described a decrease in the flicker implicit times during adaptation to high light levels in humans, which more closely reflects the changes in the current study. This decrease in implicit times or phases in the flicker ERG also corresponds with the decrease in b-wave latency during light adaptation (curve fitting reveals similar time constants ‘β’ for both b-wave latency and first harmonic phase) and implies that signal processing becomes faster during adaptation to photopic conditions.

### 3.4. A-to-B-Wave Ratio

We further analyzed the ratio between a-wave and b-wave amplitudes, to see if the amplitudes of the two components increase similarly during dark adaptation. However, it has to be considered that the b-wave was measured from the trough of the a-wave, and thus that part of its amplitude increase must be attributed to the a-wave amplitude increase. The ratio increased strongly during the first 30 min of dark adaptation, indicating that the a-wave does indeed increase more strongly than the b-wave. Since the a-wave probably originates in the photoreceptors and in the OFF-bipolar cells [41,42], whereas the b-wave mainly reflects ON-bipolar cell activity [43,44,45], such findings may suggest the presence of additional adaptation processes at the synapses between photoreceptors and ON-bipolar cells, which counteract adaptational changes at the photoreceptor level and therefore enable finer adjustments of retinal processes for adaptation.

### 3.5. Effect of Different Intensities of Adapting Light on the Dynamics of Light Adaptation

We recorded flash ERGs during light adaptation with two different light adapting backgrounds and adapting times (see Figure 7). We observed only a small impact of intensity and exposure time on the dynamics of light adaptation. Statistical analyses of the evaluated parameters revealed no significant differences for the two protocols. Nevertheless, the amplitude of the second OP peak did show divergent trends during light adaptation for the two groups (i.e., exponential and linear growth, respectively; see Figure 2). Furthermore, the recordings that were done with the 25 cd/m^2^ background showed smaller values for the b-wave latency (see Figure 1). All things considered, the strength of the adapting light had not much influence on the progress of light adaptation. Nevertheless, we propose employing a lower adapting background with a longer exposure time (i.e., protocol 1; 25 cd/m^2^ for 15 min) for the examination of light adaptation changes for flash ERG due to the additional amount of available data. Moreover, light adaptation changes appear to be completed after 10 min of light adaptation, which means that the use of a shorter, higher adaptation regime may not reveal the full extent of the changes. After 10 min, most components of the ERG did not change any further—even if the animal was exposed later on to the adapting light. Therefore we assume that the retina will be fully light adapted after 10 min which corroborates with the results of Peachey et al. from 1993 [26]. However, Bui and Fortune found the process of light adaptation to be completed only after 12–20 min in pigmented rats [29]. 

### 3.6. Effect of Different Intensities of Adapting Light on the Dynamics of Dark Adaptation

The dark adaptation effects seemed to be affected to an even lesser extent by the intensity and duration of the light adaptation state preceding it. With respect to the small impact of the adapting light, we propose to use the protocol with the intense but short light adaptation (40 cd/m^2^, 5.5 min) for examinations of the dark adaptation progress. This protocol provided a much longer period for observing the dynamics of dark adaptation. The consequence of this proposal is that light and dark adaptation cannot be studied in one session without extending the time of anesthesia in the animals.

### 3.7. Rod and Cone Selectivity of the ERGs

Although the ERG responses show that they are dominated by rod or cone signals, it cannot be excluded that both photoreceptor types contribute. Therefore, it would be of great interest to ensure that the responses are exclusively mediated by rod or cone activity. Heikkinen et al. [14] isolated cone-mediated responses by saturating rod responses using a paired-flash paradigm, whereas Hetling and Pepperberg used the paired-flash paradigm to isolate rod responses [32]. Another way to examine the effect of adaptation on rod- and cone-based ERG recordings can be obtained by using mouse models, which have only one functional photoreceptor type (i.e., rods or cones) [46,47]. However, the mutations lead to structural changes in the retina which probably also affect the ERG recordings. A third possibility to examine rod- and cone-mediated responses separately would be by using the silent substitution method, in animals that have L/M cone pigments with long wavelength-shifted absorption spectra [1,40,48,49]. In contrast to the other mouse mutants, these animals can be considered to be physiologically normal. Although the silent substitution method enables the complete isolation of rod and cone responses in LIAIS mice [50], it is not possible to use the method with completely dark backgrounds.

## 4. Methods

### 4.1. Experimental Animals

All animal experiments were performed in accordance with the principles regarding the care and use of animals adopted by the Association for Research in Vision and Ophthalmology and the Society for Neuroscience, and complied with the guidelines for the welfare of experimental animals issued by the Federal Government of Germany and the University of Erlangen–Nürnberg. Consent to conduct the experiment was also received from the local ethics authorities (Regierungspräsidium Mittelfranken Ansbach; AZ: 54-2532.1-25/13). All measurements were done on wildtype Opn1lw^LIAIS^ mice in a C57Bl/6 background, kept on a 12/12-h light/dark cycle, where they were housed. Food and water were given *ad libitum*. In total, 16 male mice (6–8 months old) were used in the present study.

### 4.2. Preparation

The mice were dark adapted overnight, prior to being prepared for the experiments. All further handling was performed under dim red illumination so that the animals largely remained dark adapted. The animals were anesthetized by an intramuscular injection of 50 mg/kg ketamine (Ketavet^®^, Pfizer, Karlsruhe, Germany) and 10 mg/kg xylazine (Rompun^®^ 2%, Bayer AG, Leverkusen, Germany). They received a subcutaneous injection of saline (10 mL/kg, 0.9%) to minimize dehydration whilst under anesthesia, and their pupils were dilated with a drop of tropicamide (Mydriaticum Stulln^®^, 5 mg/mL, Pharma Stulln, Stulln, Germany) and phenylephrin–hydrochloride (Neosynephrin POS^®^ 5%, Ursapharm, Saarbrücken, Germany). During ERG recordings, the animals were placed on a heated platform in front of the Ganzfeld stimulator. Needle electrodes placed subcutaneously at the base of the tail and at the forehead served as ground and reference electrodes, respectively. Two contact lens electrodes (Mayo Corporation, Inazawa, Japan) loaded with Corneregel^®^ (Dr. Mann Pharma, Berlin, Germany) were placed over each eye and served as active electrodes. Once the electrodes were in place, the platform was slid into the Ganzfeld bowl and the mice were allowed to readapt for at least 2 min to ensure maximal inner retinal sensitivity before recordings commenced.

Two flash ERG recording and stimulus protocols were employed. Furthermore, one flicker ERG protocol was used. The protocols are described below. Each protocol was conducted in separate sessions at least one week apart, and lasted up to approximately an hour, after which the animals were allowed to wake up.

### 4.3. Visual Stimuli

**Figure 6:**
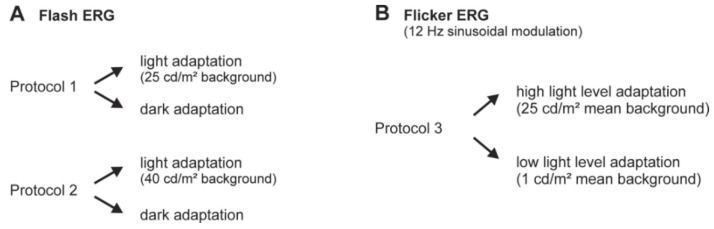
Scheme of the experimental procedures. The study consisted of two major parts: recordings with a conventional short-flash ERG (6.3 cd.s/m^2^ flash strengths) (**A**) and with a sine wave modulation (**B**). The short-flash ERGs were performed with two separate protocols, which differed in the intensity of the adapting light. Each of the protocols consisted of a light and a dark adaptation period. (**B**) The flicker ERG response to sine-wave luminance modulation was divided into adaptation periods for high (25 cd/m^2^) and low (1 cd/m^2^) mean luminances.

**Figure 7:**
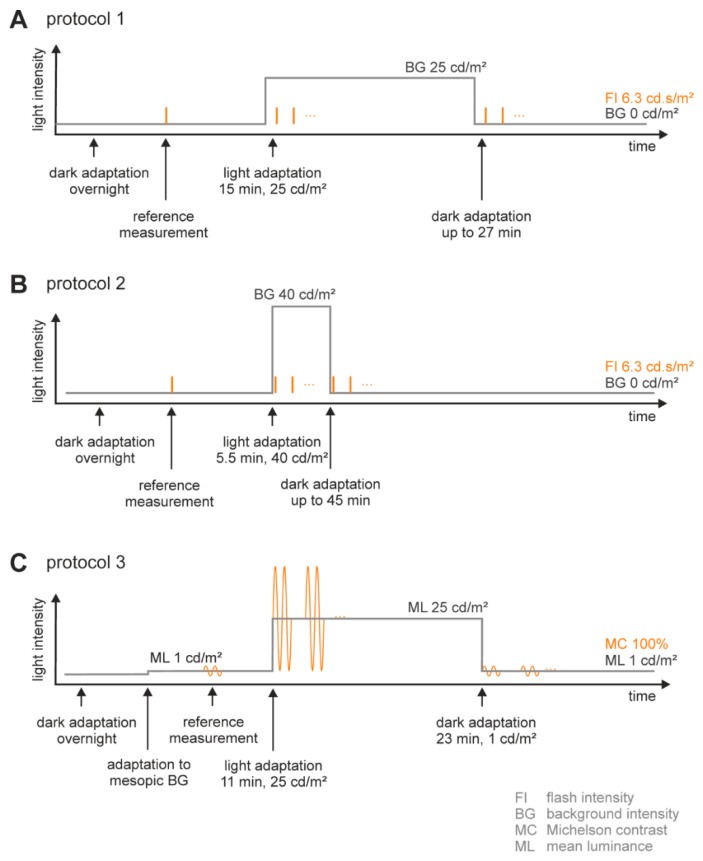
Schemes of the different adaptation protocols. (**A**) A baseline (reference) flash ERG. Flash ERGs were measured at dark adaptation. Subsequently, flash ERGs were measured at regular intervals during a 15 min period with 25 cd/m^2^ background, followed by a maximum of 27 min of dark adaptation. In the second protocol (**B**), after the reference measurement, the animals were adapted for 5.5 min to 40 cd/m^2^ followed by a maximum period of 44 min of dark adaptation; (**C**) For the flicker ERGs, the adaptation sequence was as follows: reference measurements at 1 cd/m^2^; 11 min 25 cd/m^2^; 23 min 1 cd/m^2^.

The stimuli and the adaptation lights were created by a Ganzfeld stimulator (Q450 SC, Roland Consult, Brandenburg, Germany) that was controlled by a RetiPort system (Roland Consult). We employed two different types of stimuli for flash ERGs and ERGs to sinusoidal luminance modulation, each with distinct adaptation procedures (see Figure 6 and Figure 7). 

#### 4.3.1. Flash ERGs

##### Adaptation Procedure (Figure 7A,B)

Animals were dark adapted for at least 5 min before the recordings started. A baseline recording was performed in the dark adapted state. Then, a white adaptation light of either 25 cd/m^2^ for 15 min (Figure 6A Protocol 1; Figure 7A), or 40 cd/m^2^ for 5.5 min (Figure 6A Protocol 2; Figure 7B) was presented. After the light adaptation period, the animals were dark adapted again for at least 23 min. During both light and subsequent dark adaptations, ERG responses were measured at regular instervals. Note that the shorter period of photopic adaptation in protocol 2 allowed a longer subsequent dark adaptation period to be tracked since the anesthesia lasts approximately 1 h and we did not want to reinject the animals because of the higher risk of the animal’s death.

##### Stimulation Protocol (Figure 7A,B)

ERG responses to short (maximally 5 ms), 6.3 phot cd·s/m^2^ (10.2 scot cd·s/m^2^ see Table 4) flashes of white light were recorded to track light/dark adaptation changes. The baseline measurement of each protocol was an average response from 8 consecutive flashes, recorded with a 20 s inter-stimulus interval (ISI) after the initial 5 min dark adaptation. During adaptation to photopic white light, responses to 20 flash presentations with 0.5 s ISI were measured and averaged. The measurements started immediately after the onset of the adapting light and were repeated every 60 s.

During the subsequent dark adaptation period, the same flashes were presented, but with an ISI of 20 s. When the white adapting light, prior to the dark adaptation period, was 25 cd/m^2^ (protocol 1), responses to two flashes were recorded and averaged. The measurements were repeated every 30 s (first measurement directly after offset of the white background) during the first 4 min. Thereafter, the responses to four flashes were averaged and recorded every 60 s. After the 40 cd/m^2^ white adaptation (protocol 2), responses to one flash were measured every 60 s (first measurement started 5 s after offset of the white background) for the first 4 min, followed by a period where the averaged responses to two flashes were measured every 120 s.

In a separate control experiment, we repeated the baseline measurements at four time points, with an interval of 2 min, on two animals (i.e., over a six-minute period). The ERG responses did not change strongly, indicating that the recordings were stable and any change in ERGs afterwards were indeed caused by the adaptation paradigms.

#### 4.3.2. Flicker ERGs (Figure 7C)

The influence of adaptation, under photopic and mesopic conditions, on ERG responses to sine-wave luminance modulation (white light; 12 Hz; 100% Michelson contrast) were examined in protocol 3 (see Figure 6B and Figure 7C). For this protocol, a fully dark adapted state was not possible, given the use of sinusoidal modulation around a mean luminance, rather than pulsed stimuli upon a background. Hence, a mean background luminance of 1 cd/m^2^ was switched on for 5 min, before the ERG response to 40 s sine-wave stimuli (i.e., 40 sweeps) was measured as the baseline response. Then, the mean luminance was increased to 25 cd/m^2^, and 20 flicker ERG sweeps were recorded every 30 s for 4 min, starting 10 s after the mean luminance change. Between 4 to 11 min of light adaptation, 40 flicker ERG sweeps were measured every 60 s. Then, the mean luminance was decreased to 1 cd/m^2^ again, and flicker ERGs were tracked every 60 s from 10 s, after the change in mean luminance, for a total of 23 min (40 sweeps). In every flicker ERG measurement, the first two seconds of ERG responses were not recorded, to avoid onset artefacts.

Similar to the flash ERG, we validated, in a separate control experiment, that the baseline measurement was stable, by repeating the baseline measurement at four different time points with an interval of 2 min (n = 2).

### 4.4. Signal Acquisition and Analysis

Signal acquisition was controlled by the RetiPort system. The responses were amplified 10,000× and digitized at 2048 Hz for the flash ERGs and at 1024 Hz for the flicker ERGs. Flash and flicker ERG data were analyzed with custom written MatLab (The Math Works) programs.

**Figure 8:**
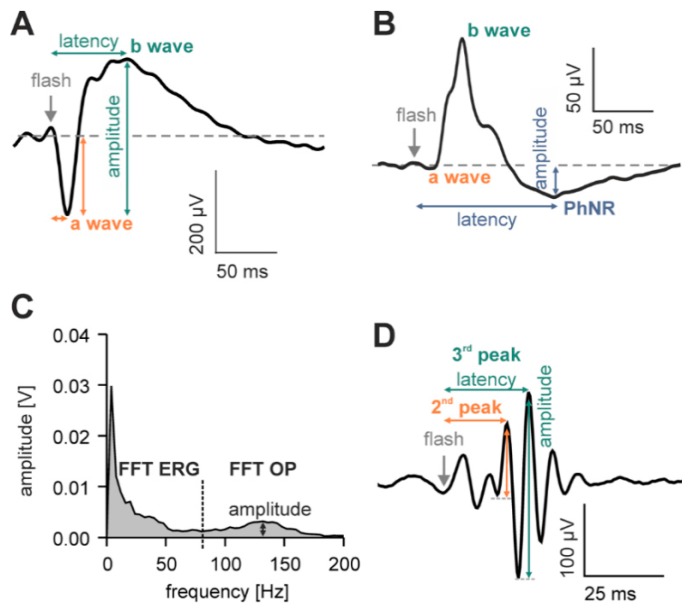
Analysis of the different flash ERG components. (**A**) Example of a scotopic flash ERG. The a-wave amplitude was defined as the absolute difference between the pre-stimulus baseline (horizontal dotted line) to the minimum of the a-wave. The difference between the a-wave trough and the peak of the b-wave was taken as the b-wave amplitude. The implicit times of the a- and b-waves were measured from the flash onset (vertical dashed line); (**B**) The photopic flash ERG contained the PhNR as a further component, in addition to the a- and the b-waves. The absolute difference between the pre-stimulus baseline and the minimum of the PhNR was taken as the PhNR amplitude; (**C**) The amplitude plot of the flash ERG in the frequency domain after the Fourier transform. The corresponding phase plot is not shown. The signal is built from a low frequency portion, containing the conventional ERG without OPs and a high frequency portion containing the OPs; (**D**) OPs in the time domain are obtained by an inverse Fourier transform of the high frequency portion. For a more detailed exploration of the OPs, the amplitudes and implicit times of the second and third OP peak were also analyzed. The differences between the second or third peak and the previous trough were taken as amplitudes of the second or third OP peak, respectively.

For flash ERGs, the oscillatory potentials (OPs) were filtered out of the leading edge of the scotopic (Figure 8A) and photopic b-waves (Figure 8B) using a variable filter paradigm, described previously [51]. Briefly, the signals were Fourier transformed into the frequency domain (Figure 8C), after which low and high frequency portions (divided by a minimum around 70 Hz; vertical dotted line in Figure 8C), representing the conventional ERG and the OPs, respectively, were identified. The low frequency portion (amplitudes at frequencies above the minimum were set to zero) was then inverse Fourier transformed to obtain an a-/b-wave complex. From this waveform, the amplitudes and implicit times of the a- and b-waves and the photopic negative response (PhNR) were measured using the usual conventions (see Figure 8A,B). OP amplitudes were defined as the maximum in the amplitude plot in the high frequency portion of the Fourier spectrum (Figure 8C). Additionally, the OPs were also analyzed in terms of the amplitudes (peak-to-peak) and latencies of the second and third peaks (Figure 8D), following inverse Fourier transform into the time domain (Figure 8D).

Flicker ERG data were also Fourier transformed to extract the amplitude and phase values of the first harmonic (fundamental) component. Noise amplitude was defined as the average of the amplitudes at a stimulus frequency of ±1 Hz. The amplitude of the first harmonic was corrected for noise by substracting the noise amplitude. If the amplitude of the first harmonic was smaller than twice the noise amplitude (i.e., the signal-to-noise ratio was smaller than 2), the response data were excluded from further analysis.

All further analyses were done with custom written Excel spreadsheets (Microsoft Corporation).

### 4.5. Statistics

Statistical analyses were performed with SPSS (SPSS 21, IBM). Since the data were not normally distributed (Shapiro–Wilk test), a Friedmann-test was used to analyze alterations in the different ERG parameters during adaptation to either a higher or lower light level. To analyze if the two flash-ERG adaptation protocols had any effects on the various ERG components, we compared (i) the amplitudes and implicit times at time point t = 0 min and (ii) the change of the parameters during the first 5 min for light adaptation and the first 26 min of dark adaptation, with a Mann–Whitney-U test. An alpha value of 0.05 was adopted as the definition threshold for significance, but the significance level for each protocol was Bonferroni corrected for multiple testing, by dividing the significance value by the number of tests. Additionally, we performed curve fittings on all ERG parameters for protocols 1 and 2 with one of the following functions:

y(t) = y_0_ + α(1 − e^−βt^) (1)

exponential rise/fall to maximum/minimum.

y(t) = δ + αe^βt^ (2)

exponential growth/decrease.

y(t) = y_0_ + εt (3)

linear increase/decrease.

## 5. Conclusions

In conclusion, we found significant changes for most ERG components during light and during dark adaptation. We also found the a-wave to increase more strongly during dark adaptation than the b-wave, suggesting that additional adaptational processes may be present at the photoreceptor synapse. Furthermore, the variable adaptation effects on the individual OP wavelets further supports the notion that they originate from different cell populations and actions. Additionally, we tested the effect of two different settings of adapting light and did not find a strong effect on either light or subsequent dark adaptation. Our ERG recording protocols thus provide the possibility to examine the complex processes of adaptation in vivo and non-invasively. These protocols could be used in future studies to investigate the impact of retinal cells or proteins in adaptational processes in mouse models of retinal disorders. Furthermore, this study approaches changes in flash and flicker ERGs during light and dark adaptation in detail to provide a comprehensive basis for comparison of future investigations.

## Figures and Tables

**Table 1:** Summary statistics of changes in ERG components during light adaptation. All data shown in Figure 1C and Figure 2C were tested with a Friedmann-test, which considers all measured time points, while the animal was light adapted. Significance levels were corrected via the Bonferroni method: α_Bonferroni_ = 0.005. *p*-values that were below this significance level are highlighted.

ERG Component		Protocol	% Change During Light Adaptation Mean ± SD	*p*-Value
b-wave	amplitude [µV]	25 cd/m^2^	+21.8 ±18.8	**<0.0005**
		40 cd/m^2^	+22.3 ± 22.4	0.007 ^1^
	implicit time [ms]	25 cd/m^2^	−7.7 ± 4.2	**<0.0005**
		40 cd/m^2^	−4.8 ± 1.8	**<0.0005**
PhNR	amplitude [µV]	25 cd/m^2^	+85.2 ± 116.0	0.007 ^1^
		40 cd/m^2^	+47.5 ± 228.1	0.766
	implicit time [ms]	25 cd/m^2^	−8.5 ± 12.5	0.722
		40 cd/m^2^	+5.6 ± 13.3	0.722
2nd oscillatory potential (OP) peak	amplitude [µV]	25 cd/m^2^	+101.6 ± 103.9	0.453
		40 cd/m^2^	+133.9 ± 184.2	0.066
	implicit time [ms]	25 cd/m^2^	−1.7 ± 1.7	**0.002**
		40 cd/m^2^	−1.2 ± 2.2	0.140
3rd OP peak	amplitude [µV]	25 cd/m^2^	+70.6 ± 57.9	**<0.0005**
		40 cd/m^2^	+102.9 ± 127.7	0.017 ^1^
	implicit time [ms]	25 cd/m^2^	−5.3 ± 1.8	**<0.0005**
		40 cd/m^2^	+0.7 ± 2.5	**0.002**
all OP (FFT analysis)	amplitude [µV]	25 cd/m^2^	+40.8 ± 47.4	0.008 ^1^
		40 cd/m^2^	+29.2 ± 25.1	0.131

^1^ *p*-value was not significant after Bonferroni correction for multiple testing.Highlighted *p*-values are below significance level.

**Table 2:** Summary of statistical evaluation of the changes of ERG components during dark adaptation. A Friedmann-test was performed to analyze the increase/decrease of the parameters during dark adaptation. Significance level was corrected via the Bonferroni method: α_Bonferroni_ = 0.005. *p*-Values that were significant after the Bonferroni correction are highlighted.

ERG Component		Protocol	% Change during Light Adaptation Mean ± SD	*p*-Value
a-wave	amplitude [µV]	25 cd/m^2^	+309.3 ± 50.6	**<0.0005**
		40 cd/m^2^	+840.4 ± 571.0	**<0.0005**
	implicit time [ms]	25 cd/m^2^	+9.2 ± 6.6	0.328
		40 cd/m^2^	−7.0 ± 14.2	**0.004**
b-wave	amplitude [µV]	25 cd/m^2^	+100.0 ± 38.7	**<0.0005**
		40 cd/m^2^	+126.8 ± 64.3	**<0.0005**
	implicit time [ms]	25 cd/m^2^	+12.2 ± 9.2	0.867
		40 cd/m^2^	−17.0 ± 13.7	0.52
a-to-b ratio		25 cd/m^2^	+114.3 ± 45.6	**<0.0005**
		40 cd/m^2^	+301.9 ± 277.2	**<0.0005**
2nd OP peak	amplitude [µV]	25 cd/m^2^	+321.9 ± 76.2	**<0.0005**
		40 cd/m^2^	+588.2 ± 240.0	**<0.0005**
	implicit time [ms]	25 cd/m^2^	+0.1 ± 8.3	0.006 ^1^
		40 cd/m^2^	−2.8 ± 3.2	0.0061
3rd OP peak	amplitude [µV]	25 cd/m^2^	+272.2 ± 85.5	**<0.0005**
		40 cd/m^2^	+435.0 ± 170.6	0.0061
	implicit time [ms]	25 cd/m^2^	−2.5 ± 4.6	**<0.0005**
		40 cd/m^2^	+1.7 ± 4.4	**<0.0005**
all OP	amplitude [µV]	25 cd/m^2^	+78.9 ± 55.7	0.008 ^1^
(FFT analysis)		40 cd/m^2^	+124.4 ± 57.5	**<0.0005**

^1^ *p*-value was below significance level after Bonferroni correction for multiple testing. Highlighted *p*-values are below significance level.

**Table 3:** Summary of the first harmonic analyses. Changes for the amplitude and phase of the photopic first harmonic were tested for significant changes during light adaptation with a Friedmann-test. Due to the bad-signal-to noise ratio, some of the data for the dark adaptation had to be excluded. Therefore no statistical tests could be performed on the mesopic data. Significance level was corrected via the Bonferroni method: α_Bonferroni_ = 0.025. *p*-Values that were significant after the Bonferroni correction are highlighted.

1st Harmonic Parameter	Mean Luminance	% change During Light Adaptation Mean ± SD	*p*-Value	Mean Luminance	%-Change During Dark Adaptation Mean ± SD
amplitude [µV]	25 cd/m^2^	−5.8 ± 39.3	**0.002**	1 cd/m^2^	+438.1 ± 304.4
phase [deg]	25 cd/m^2^	−8.7 ± 7.2	**<0.0005**	1 cd/m^2^	+51.1 ± 159.2

Highlighted *p*-values are below significance level.

**Table 4:** Luminances used in the study. Conversion factor from photopic (phot) cd/m^2^ to scotopic (scot) cd/m^2^, and from phot cd·s/m^2^ to scot cd·s/m^2^ is 1.624.

	Phot cd/m^2^	Log Phot cd/m^2^	Scot cd/m^2^	Log Scot cd/m^2^
Background light	1.0	0.0	1.6	0.2	
	25.0	1.4	40.6	1.6	
	40.0	1.6	65.0	1.8	
	**Phot cd·s/m^2^**	**Log Phot cd·s/m^2^**	**Scot cd·s/m^2^**	**Log Scot cd·s/m^2^**
Flash	6.3	0.8	10.2	1.0

## Supplementary Materials

The following are available online at www.mdpi.com/2411-5150/1/4/23/s1. Table S1: Summary of the parameters fits for the light adaptation flash data; Table S2: Statistical analyses of the differences of the parameter calue y(t_0_) and the change of the parameters during the first 5 min of light adaptation between the two different protocols; Table S3: Highlighted p-values are below the significance level; Table S4: Statistical analyses of the differences of the parameter value y(t_0_) and the change of the parameters during the first 26 min of light adaptation between the two different protocols; Table S5: Summary of fitting parameters for the flicker 1st harmonic.
