# Supplementary material for: Electrophysiological Studies on The Dynamics of Luminance Adaptation in the Mouse Retina
Source: Vision (Basel). 2017 Oct 17;1(4):23. doi: 10.3390/vision1040023 (PMC6835873; doi:10.3390/vision1040023)
Supplement: Supplementary file 1 [file vision-01-00023-s001.docx]

Electrophysiological Studies On The Dynamics Of Luminance Adaptation In The Mouse Retina

Anneka Joachimsthaler ^1,2^, Tina I. Tsai ^1,2^, Jan Kremers ^1,^*

^1^ Department of Ophthamlology, University Hospital Erlangen, 91054 Erlangen, Germany; anneka.joachimsthaler@uk-erlangen.de (A.J.); tina.tsai@uk-erlangen.de (T.I.T.)

^2^ Department of Biology, Animal Physiology, FAU Erlangen-Nürnberg, 91058 Erlangen, Germany

***** Correspondence: Jan.Kremers@uk-erlangen.de

Supplementary data

**T**able S1: Summary of the parameters fits for the light adaptation flash data. The fitting parameters for data of protocol 1 have a gray background; the data without a background stem from protocol 2. Units of the different parameters are given for amplitude and latency in the table heading.

| **ERG component** | | **Fitting function** | **y_0_**  **[µV]; [ms]** | **α**  **[µV*min^-1^]; [ms*min^-1^]** | **β**  **[min^-1^]** | **ε**  **[µV*min^-1^]; [ms*min^-1^]** | |
| --- | --- | --- | --- | --- | --- | --- | --- |
| b-wave | amplitude | y(t) = y_0_ + α (1 – e^-βt^) | 95.8 | 25.7 | 0.296 | |  |
|  |  |  | 87.2 | 4267.7^1^ | 0.001^1^ | |  |
|  | latency | y(t) = y_0_ + α (1 – e^-βt^) | 42.8 | -3.6 | 0.152 | |  |
|  |  |  | 45.1 | -2.8 | 0.341 | |  |
| PhNR | amplitude | y(t) = y_0_ + α (1 – e^-βt^) | 15.5 | 14.3 | 0.166 | |  |
|  |  |  | 26.1 | 42.0 | 0.272 | |  |
|  | latency | y(t) = y_0_ + εt | 156.2 |  |  | | -0.905 |
|  |  |  | 166.5 |  |  | | -1.425 |
| 2^nd^ OP peak | amplitude | y(t) = y_0_ + α (1 – e^-βt^) | 7.4 | 3.9 | 0.658 | |  |
|  |  |  | 1.6 | 1817.7^1^ | 0.001^1^ | |  |
|  | latency | y(t) = y_0_ + α (1 – e^-βt^) | 62.6 | -2.7 | 0.093 | |  |
|  |  |  | 62.9 | -1.4 | 0.278 | |  |
| 3^rd^ OP peak | amplitude | y(t) = y_0_ + εt | 20.5 |  |  | | 0.643 |
|  |  |  | 10.6 |  |  | | 1.615 |
|  | latency | y(t) = y_0_ + εt | 72.2 | -0.9 | 0.414 | |  |
|  |  |  | 71.3 | -5.1 | 0.096 | |  |
| all OP  (FFT analysis) | amplitude | y(t) = y_0_ + α (1 – e^-βt^) | 0.4 | 0.1 | 0.609 | |  |
|  |  |  | 0.5 | 0.2 | 0.450 | |  |

^1^ Response-adaptation profiles showed variable trends for the different animals. Therefore the fitting of the data was difficult and the fitting parameters are unlikely.

**T**able S2: Statistical analyses of the differences of the parameter value y(t_0_) and the change of the parameters during the first 5 min of light adaptation between the two different protocols. The data of protocol 1 (25 cd/m²) have a gray background. The data without a background stem from protocol 2. Units of the parameters are given for amplitude and latency in the table heading. The significance level was corrected after Bonferroni (nBonferroni=9).

| **ERG component** | | **background intensity** | **y(t_0_)[µV]; [ms]** | | **y(t_5_)-y(t_0_) [µV]; [ms]** | |
| --- | --- | --- | --- | --- | --- | --- |
|  |  |  | **mean ± sd** | **p-value** | **mean ± sd** | **p-value** |
| b-wave | amplitude | 25 cd/m² | 97.5 ± 28.1 | 0.721 | 27.0 ± 8.6 | 0.959 |
|  |  | 40 cd/m² | 87.9 ±12.3 |  | 18.6 ± 18.3 |  |
|  | latency | 25 cd/m² | 43.0 ± 1.7 | 0.028^1^ | -2.2 ± 1.0 | 0.798 |
|  |  | 40 cd/m² | 45.1 ± 1.6 |  | -2.2 ± 0.9 |  |
| PhNR | amplitude | 25 cd/m² | 18.4 ± 16.7 | 0.234 | 10.6 ± 17.0 | 0.328 |
|  |  | 40 cd/m² | 27.0 ± 16.2 |  | 31.0 ± 38.6 |  |
|  | latency | 25 cd/m² | 161.1 ± 26.2 | 0.422 | -12.6 ± 35.2 | 0.721 |
|  |  | 40 cd/m² | 168.2 ± 23.5 |  | -8.3 ± 21.4 |  |
| 2^nd^ OP peak | amplitude | 25 cd/m² | 7.2 ± 5.3 | 0.613 | 4.1 ± 5.1 | 0.867 |
|  |  | 40 cd/m² | 5.7 ± 5.0 |  | 3.0 ± 7.7 |  |
|  | latency | 25 cd/m² | 61.6 ± 1.0 | 0.463 | 0.2 ± 1.3 | 0.232 |
|  |  | 40 cd/m² | 61.2 ± 1.0 |  | 0.7 ± 1.3 |  |
| 3^rd^ OP peak | amplitude | 25 cd/m² | 18.0 ± 11.0 | 0.074 | 5.8 ± 11.4 | 0.888 |
|  |  | 40 cd/m² | 11.7 ± 6.8 |  | 7.5 ± 11.2 |  |
|  | latency | 25 cd/m² | 71.4 ± 1.1 | 0.222 | -2.1 ± 0.6 | 0.045^1^ |
|  |  | 40 cd/m² | 72.1 ± 1.3 |  | -0.5 ± 1.8 |  |
| all OP  (FFT analysis) | amplitude | 25 cd/m² | 0.5 ± 0.4 | 0.743 | 0.2 ± 0.3 | 0.236 |
|  |  | 40 cd/m² | 0.4 ± 0.1 |  | 0.1 ± 0.1 |  |

^1^ p-value was below significance level before Bonferroni correction

**T**able S3: Summary of the fitting parameters for the dark adaptation flash data. The fitting parameters for data of protocol 1 have a gray background; the data without a background stem from protocol 2.. Units of the different parameters are given for amplitude and latency in the table heading.

| **ERG component** | | **Fitting function** | **y_0_ or δ [µV]; [ms]** | **α [µV*min^-1^]; [ms*min^-1^]** | **β [min^-1^]** | **ε [µV*min^-1^]; [ms*min^-1^]** |
| --- | --- | --- | --- | --- | --- | --- |
| a-wave | amplitude | y(t) = y_0_ + α (1 - e^-βt^) | 46.9 | 41059.5^1^ | 0.000^1^ |  |
|  |  |  | 17.1 | 402.6 | 0.029 |  |
|  | latency | y(t) = y_0_ + α (1 - e^-βt^) | 13.8 | 2.4 | 1.311 |  |
|  |  |  | 17.1 | -6.8 | 0.008 |  |
| b-wave | amplitude | y(t) = y_0_ + εt | 155.7 |  |  | 6.917 |
|  |  |  | 181.6 |  |  | 7.278 |
|  | latency | y(t) = y_0_ + εt | 41.0 |  |  | 0.227 |
|  |  |  | 46.2 |  |  | -0.032 |
| a-to-b ratio |  | y(t) = y_0_ + α (1 - e^-βt^) | 0.3 | 0.6 | 0.039 |  |
|  |  |  | 0.2 | 0.5 | 0.072 |  |
| 2^nd^ OP peak | amplitude | y(t) = y_0_ + εt | 18.3 |  |  | 4.002 |
|  |  |  | 7.9 |  |  | 3.287 |
|  | latency | y(t) = y_0_ + εt | 55.1 |  |  | -0.092 |
|  |  |  | 55.3 |  |  | -0.076 |
| 3^rd^ OP peak | amplitude | y(t) = δ + αe^βt^ | -5.6 | 35.1 | 0.059 |  |
|  |  |  | -7.0 | 36.8 | 0.040 |  |
|  | latency | y(t) = y_0_ + α (1 - e^-βt^) | 66.7 | -10.1 | 0.024 |  |
|  |  |  | 78.6 | -17.5 | 0.109 |  |
| all OP  (FFT analysis) | amplitude | y(t) = δ + αe^βt^ | 0.7 | 0.1 | 0.081 |  |
|  |  |  | 0.5 | 0.3 | 0.041 |  |

^1^ Response-adaptation profiles showed variable trends for the different animals. Therefore the fitting of the data was difficult and the resulting fitting parameters are unlikely.

**T**able S4: Statistical analyses of the differences of the parameter value y(t_0_) and the change of the parameters during the first 26 min of light adaptation between the two different protocols. The data of protocol 1 (25 cd/m²) have a gray background; the data without a background stem from protocol 2. . Units of the parameters are given for amplitude and latency in the table heading. The significance level was corrected after Bonferroni (nBonferroni=9).

| **ERG component** | | **background intensity** | **y(t_0_) [µV]; [ms]** | | **y(t_26_)-y(t_0_)**  **[µV]; [ms]** | |
| --- | --- | --- | --- | --- | --- | --- |
|  |  |  | **mean ± sd** | **p-value** | **mean ± sd** | **p-value** |
| a-wave | amplitude | 25 cd/m² | 47.3 ± 16.0 | 0.724 | 180.3 ± 45.4 | 0.368 |
|  |  | 40 cd/m² | 43.8 ± 38.7 |  | 201.5 ± 70.2 |  |
|  | latency | 25 cd/m² | 14.3 ± 3.9 | 0.435 | 2.6 ± 4.0 | 0.368 |
|  |  | 40 cd/m² | 15.9 ± 2.6 |  | 0.1 ± 2.9 |  |
| b-wave | amplitude | 25 cd/m² | 167.1 ± 31.6 | 0.435 | 148.0 ± 85.3 | 0.683 |
|  |  | 40 cd/m² | 184.9 ± 47.8 |  | 193.7 ± 80.5 |  |
|  | latency | 25 cd/m² | 42.5 ± 4.8 | 0.435 | 6.2 ± 8.2 | 0.214 |
|  |  | 40 cd/m² | 45.0 ± 6.4 |  | 0.0 ± 5.9 |  |
| a-to-b ratio |  | 25 cd/m² | 0.3 ± 0.1 | 0.724 | 0.4 ± 0.1 | 0.933 |
|  |  | 40 cd/m² | 0.2 ± 0.2 |  | 0.4 ± 0.2 |  |
| 2^nd^ OP peak | amplitude | 25 cd/m² | 28.8 ± 11.1 | 0.222 | 112.8 ± 21.7 | 0.109 |
|  |  | 40 cd/m² | 21.5 ± 9.9 |  | 66.2 ± 37.9 |  |
|  | latency | 25 cd/m² | 52.8 ± 2.3 | 0.284 | -0.3 ± 4.1 | 0.461 |
|  |  | 40 cd/m² | 50.6 ± 3.8 |  | 2.6 ± 3.5 |  |
| 3^rd^ OP peak | amplitude | 25 cd/m² | 43.2 ± 25.5 | 0.833 | 136.0 ± 43.9 | 0.028^1^ |
|  |  | 40 cd/m² | 40.1 ± 20.5 |  | 47.8 ± 45.6 |  |
|  | latency | 25 cd/m² | 62.8 ± 3.9 | 0.524 | 0.9 ± 5.2 | 0.283 |
|  |  | 40 cd/m² | 63.8 ± 4.6 |  | -2.6 ± 4.1 |  |
| all OP  (FFT analysis) | amplitude | 25 cd/m² | 1.0 ± 0.1 | 0.833 | 0.8 ± 0.4 | 0.283 |
|  |  | 40 cd/m² | 1.0 ± 0.3 |  | 0.4 ± 0.4 |  |

^1^ p-value was below significance level before Bonferroni correction

**T**able S5: Summary of fitting parameters for the flicker 1st harmonic. Units of the different parameters are given for amplitude and latency in the table heading.

| ERG component | | Fitting function | y_0_  [µV]; [ms] | α  [µV*min^-1^]; [ms*min^-1^] | β  [min^-1^] | ε  [µV*min^-1^]; [ms*min^-1^] |
| --- | --- | --- | --- | --- | --- | --- |
| high light level | amplitude | y(t) = y_0_ + α (1 - e^-βt^) | 0.7 | -42.9 | 0.001 |  |
|  | phase | y(t) = y_0_ + α (1 - e^-βt^) | 297.5 | -33.5 | 0.162 |  |
| low light level | amplitude | y(t) = y_0_ + αe^βt^ | 0.1 | 0.0 | 0.119 |  |
|  | phase | y(t) = y_0_ + εt | 148.6 |  |  | 1.552 |
